# Reply: Selection of controls

**DOI:** 10.1038/sj.bjc.6603976

**Published:** 2007-09-18

**Authors:** S O Dalton, M Düring, L Ross, K Carlsen, P B Mortensen, J Lynch, C Johansen

**Affiliations:** 1 Institute of Cancer Epidemiology, Danish Cancer Society, Strandboulevarden 49, Copenhagen, DK-2100, Denmark

**Sir,**

We thank von Euler-Chelpin and Lynge for their interest and comments. However, we believe their comments result from some misunderstandings. First of all, our study was not a case–control study. Secondly, and more importantly, we did not aim to investigate breast cancer incidence by socioeconomic level and this could not have been estimated from our study. Our aim was to assess, given a breast cancer, the probability of having a high-risk *vs* a low-risk case at the time of diagnosis by socioeconomic position. This we investigated in a nation-wide population-based cohort of 28 765 women diagnosed with breast cancer in Denmark between 1983 and 1999 using a wide range of socioeconomic factors such as educational level and income (Dalton *et al*, 2006).

In Denmark, as in most other Western countries, the overall incidence of breast cancer increases with higher socioeconomic position (Danø *et al*, 2003, 2004). We have shown that despite the higher levels of breast cancer incidence among more advantaged women (Danø *et al*, 2003), relatively more women with less advantaged socioeconomic position are being diagnosed with a high-risk breast cancer and as a consequence are more likely to have to go through more extensive treatment and probably suffer a worse prognosis.
